# Supplementary figures and images for: Effects of cowpea mild mottle virus on soybean cultivars in Brazil
Source: PeerJ. 2020 Aug 31;8:e9828. doi: 10.7717/peerj.9828 (PMC7469931; doi:10.7717/peerj.9828)

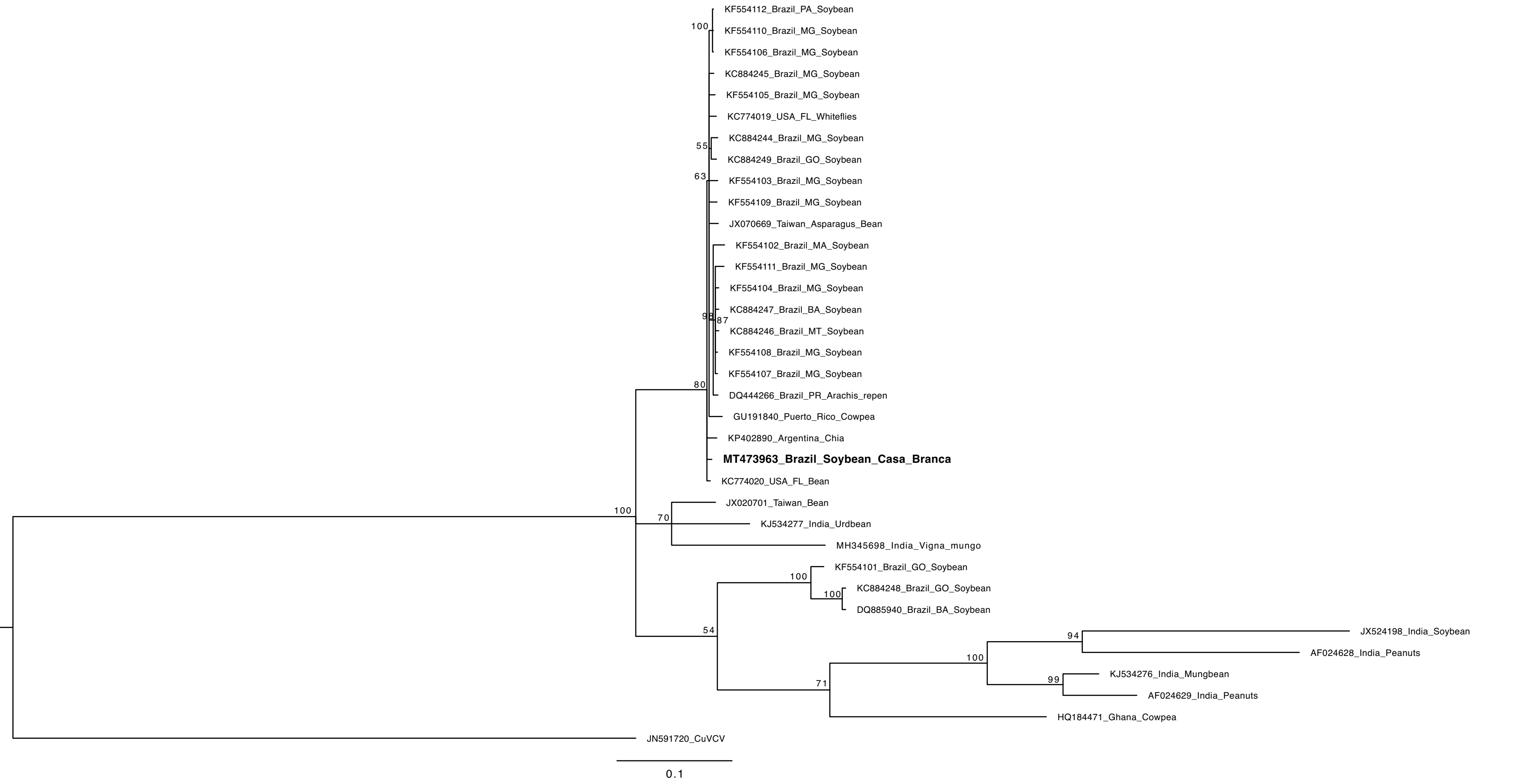

Supplement: Supplemental Information 1 — Phylogenetic tree of the nt sequences of the CPMMV coat protein (cp) GenBank using Bayesian inference (implemented in MRBAYES V. 3.1, with model GTR+I+G and 10 million generations). The analysis displayed two defined cluster. The CPMMV Brazil Soybean Casa Branca_BR isolate is included with isolates from Argentina (1), Brazil (20), India (5), Puerto Rico (1) Taiwan (2), USA (2) and Ghana (1). Cucumber vein-clearing virus (CuVCV; genus Carlavirus, family Betaflexiviridae) was used as outgroup. [file peerj-08-9828-s001.pdf]

### Botucatu - SP

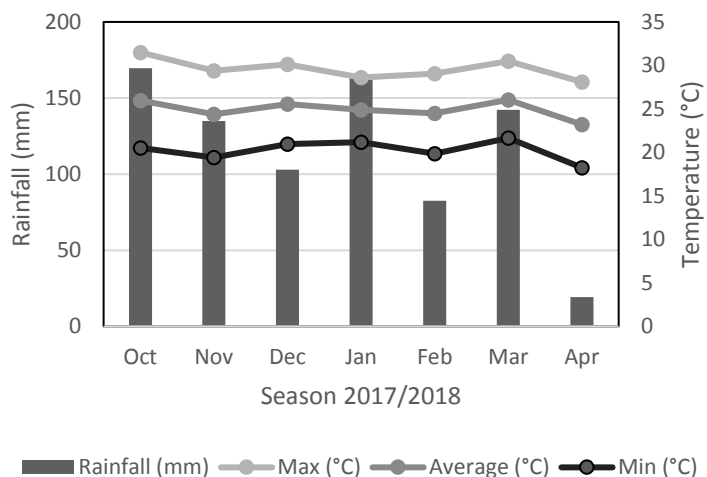

### Mogi Mirim - SP

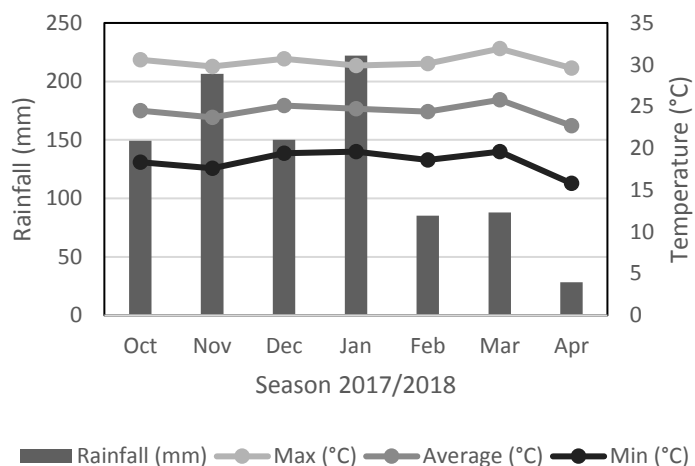

### Planaltina - DF

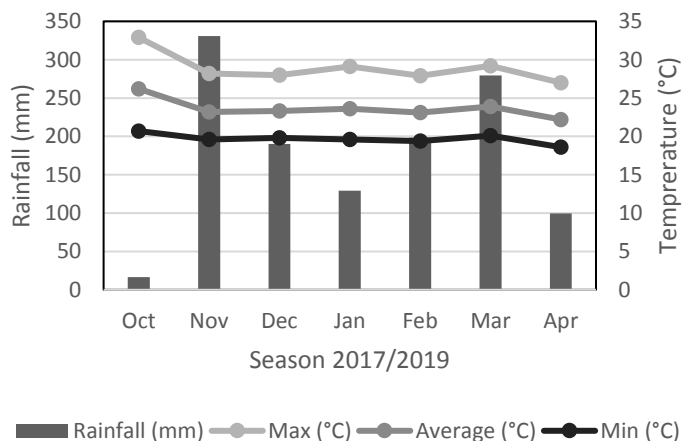

### Pedra Preta - MT

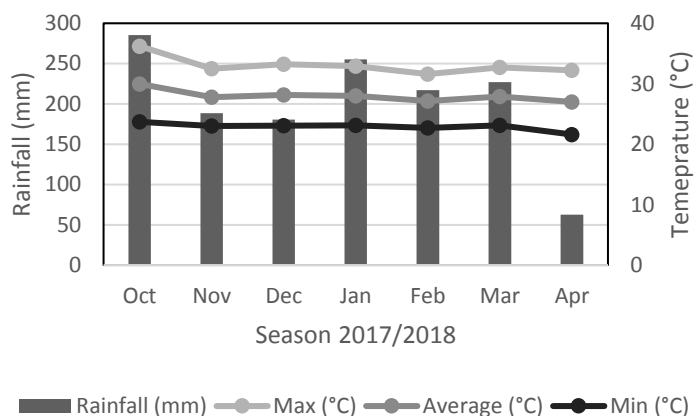

Supplement: Supplemental Information 2 — Monthly maximum, minimum and average temperature, rainfall occurring during the field experiments in all tested area (Source: Instituto Nacional de Meteorologia do Brasil - INMET). [file peerj-08-9828-s002.pdf]
